# Ethical aspects of artificial intelligence in general surgical practice

**DOI:** 10.1590/0100-6991e-20243762EDIT01-en

**Published:** 2024-05-02

**Authors:** ALBERTO R FERRERES

**Affiliations:** 1 - University of Buenos Aires, Surgery, Education and Research - Buenos Aires - Buenos Aires - Argentina; 2 - University of Washington, Surgery - Seattle - WA - Estados Unidos

## EDITORIAL

Artificial intelligence (AI) has burst into the surgical arena and has come to stay. The irruption of AI in surgical practice represents a major innovation and since no one can predict its fate, strict compliance with ethical principles and good practices is mandatory. The well-known adage “Information is power” has been replaced by the new and current paradigm: “The ability and capacity of processing data information is the real power”. So, there is a definitive role for AI to assess, analyze, compare, prioritize “big data”, defined as “information assets characterized by such a high volume, speed, and variety to require specific technology and evaluation methodology for its transformation into value”. AI is a multidisciplinary, ever-evolving field using tools to automate tasks and with the goal to replicate, improve and surpass human capabilities^1^. Machine learning (ML) is an AI-derived methodology which applies algorithms to find patterns, model relationships, and generate predictions in complex datasets^2^. While AI has the potential to optimize and transform many aspects of surgical decision-making, there are barriers and pitfalls to its integration and acceptance in the surgical field, as with new technologies. Another broad aspect of AI is the use of assisted conversational agents, (chatbox and similars) which allow a dialogue with users by receiving and processing their enquiries and replying with texts, messages or voice reply.

We, as members of the surgical community, must fight for enhancing trust and transparency in the use of AI in a responsible way. This approach requires the critical appraisal of the quantity and quality of the data being used to generate models to mitigate biases in algorithms. To fully integrate the technology and maximize its potential there should be a conscientious and proactive management of all the eventual ethical, legal and financial implications. The quality of data will influence the quality of the models and lastly, its outcomes. There seems to be apparent geographic, social, racial and ethnic biases in the current implementations of ML and these behaviors should be prevented. Both, surgeons and society must remain alert against this type of exclusion and inclusion biases, and the audit of databases is warranted to guarantee high quality data output, which is the only way AI algorithms may offer accurate predictions and adequate performance^3^. 

An appropriate framework to address the impact of AI and its byproducts in the surgical arena is providing answers to the why, the how and the what, meaning the purpose, the process of its implementation (specific actions taken to realize the why) and results of the why, represented by the final and definitive outcomes (final proof). 

**Figure 1.**
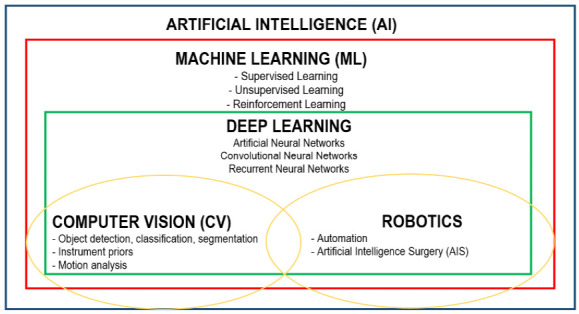

Deep learning (DL) represents a subcategory of ML and its algorithms are prepared to recognize patterns in the collected data and offer predictions, decisions or recommendations by the use of layers of artificial neural networks which resemble the human decision-making process. There are 2 additional systems within DL, represented by computer vision and robotics, as shown in Figure 1. 

AI processes may be classified in locked and adaptative ones, the first always providing the same output in response to the same input, meanwhile in the adaptative ones, the system is able to continue learning through the acquisition of new data, so they will not offer every time the same output in response to the same input, since it will consider new variables depending upon the new information acquired. Besides, AI systems may be non-autonomous, situation in which a human exerts supervision or autonomous, where the output is not supervised by a human being. In order to assess potential ethical conflicts it is important to distinguish AI methodologies: a) white-box AI system: which receives input and uses a reasoning process which is both transparent and understandable to forward an output in the form of prediction, decision or recommendation; b) black-box AI system: the system receives input and uses a reasoning process which is opaque and not understandable nor interpretable to provide an output. Deep learning algorithms belong to this category. There are additional features of black-box AI systems which are necessary knowing for a better understanding of the ethical implications. These are: a) explainability: in contrast with white-box operational systems, in black-box ones there is a huge limitation to decoding and understanding the rationale which leads the AI to predict or decide, making the process impossible to be explained or justified; b) although the human user may not understand the AI black-box process, the individual is able to independently assess the accuracy of the provided output (result); c) however, there may be situations where the individual will not be able to understand the AI black-box reasoning nor independently assess the accuracy of the final result. Conversational agents also have a role in the healthcare world, since these AI-based programs allow and engage in a dialogue with users by receiving questions or concerns and delivering their reply by text message, image or voice format.

AI allows two main trends associated to Surgery which are: AI in Surgery, related to the use of ad-hoc algorithms involved in learning for prediction and/or classification in the pre and post-operative phases; and AI-S (Artificial Intelligence Surgery), characterized by the utilization of such algorithms during a surgical procedure and covering autonomous actions during it. Essentially the goal of AI-based systems in every-day surgical care is to enhance and refine the decision-making process with different kinds of aids, tools and assistance. 

Some of key features of the application of AI in surgical care include the following:

- Predictive analysis and risk assessment based on previous outcomes;
- Intraoperative assistance with guidance and navigation. AI is particularly effective in image-guided (laparoscopic and robotic) surgery, where the screen can display information or guidance from AI-aided systems (e.g. 3-D navigation systems for performing complex liver surgery) during the operation;
- Role in surgical education and training. Based on the review of millions of surgical videos, AI will possess the ability to assist in the performance of an operation, anticipating the next 15 to 30 seconds of an operation and provide additional oversight during the surgery, or, as a navigational system. In the future, anticipation of surgical events could allow surgeons to change their courses of action, if necessary. This application would be able to make laparoscopic procedures (like cholecystectomy) safer by placing an overlay on the video screen during an operation to suggest where it is safer to guide the course of the operation. The ethical question is who is the ultimate accountable in case of a suboptimal outcome. Liability will always be on the head of the professional. From the ethical point of view, the dilemma lies about the knowledge the patient has regarding the decision-making process.

The need of an adequate balance between maximizing benefits and minimizing risks is mandatory, thus the requirement for governance from the ethical, legal and regulatory point of view^4^. The perceived idea is that AI will improve health care and surgical delivery-care systems, diminishing human biases, enhancing the accuracy of diagnostic methodologies, but probably undermining the patient-physician relationship, the deskilling of physicians, compromise of transparency, inappropriate management due to undiagnosed errors within the AI decision-making process or algorithm’s bias or mistakes. 

The main ethical issues that arise from applying AI to surgery relate to human agency, accountability for errors, technical robustness, privacy and data governance, transparency, diversity, non-discrimination, and fairness. The issue of the adequate informed consent process should be considered an ethical imperative, but also a legal mandate and as such needs to be respected and performed in clinical practice. However, AI and its byproducts may be considered nowadays a disruptive technology, but it is here to stay and on the verge of revolutionizing surgical care, in particular in settings who are capable to afford its additional initial costs and economic impact. AI will definitively have an expanding role in healthcare administration and patient care, making hospital and health care systems operations more efficient and less costly and human error-prone. Postoperative consultations with nurses and surgeons may be replaced by a chatbot answering patient’s questions and addressing their concerns. Surgery as a profession and surgeons, as ethical agents, must assure the availability of AI technologies for all kind of patients and in all settings, preventing discrimination or lack of access. 

Some of the ethical perils of AI include the accountability, trust issues and data limitations and bias. A highly predictive algorithm depends on improving the depth, quality and diversity of the data fed into the risk models. The power and accuracy of AI prediction models will depend on the acquisition of data from a diverse pool, including different practice settings (rural, community hospitals and university/ academic centers). It is important, as with any surgical tool, the need of surgeons to be educated about the pros and cons as well as the limitations of any given AI application. A key challenge is ensuring access to large amounts of patient data safely while still protecting the privacy of patient data. Data quantity and diversity determine if AI models are widely applicable and reproducible regardless of variations in patient and surgeon factors^5^.

Surgical Ethics (SE) is based on the recognition of the rights of patients undergoing or requiring surgical care, Surgery being a moral practice and the surgeon, a moral fiduciary agent. The ethical duties of a surgeon stem from these ethical foundations: the surgeons’ responsibility to Society, to Surgery as a whole, and to the self-regulation of the surgical profession; the professional obligation to use the body of scientific knowledge entrusted to surgeons to serve others and lastly, the relationship among surgeons, between surgeons and their associations, and between these two and the society to which they belong^6^. The surgeon-patient relationship intertwines the surgeon on one side, who holds the role of “authority” because of training, expertise, wisdom, and judgment; and on the other, the patient, who holds a position of “authority” to consent that upon his or her body an intervention may be performed. This bond is characterized by trust. The ethical principles in the medical field, as collated by Beauchamp and Childress, are: beneficence, non-maleficence, respect for autonomy and justice^7^.

Ethical theories are predominantly related to three schools: a) consequentialism or utilitarianism, focuses on the outcomes; b) deontology, founded by Kant and based in the categorical imperative; c) virtue ethics, based on the teachings of the classis Greek ancient philosophers and represented by the quest to understand and live a life of moral character. There have been several attempts to translate the principles of virtue ethics into a modern technology-permeated context, leading to the concept of “flourishing ethics”^8^. Its key tenets include the following concepts: a) human thriving is central to ethics, b) as social individuals, individuals can only prosper in society, c) flourishing imposes humans to do and transcend in what they are especially fitted to do, d) genuine knowledge, theoretical reasoning, autonomous acting in a fair way are required for that booming, e) in order to achieve practical reasoning, an ethical ability to deliberate about the goals and select a wise course of action is a must.

The goals pursued by the development of AI and its subsystems should include:

- Enhancement of efficiency and thus improving benefits, mostly from an economic point of view and related to the economic benefits that may arise from its use;
- Societal supervision/ surveillance, the collection of data from millions of individuals may allow the identification of patterns and behavior;
- Thriving of humanity, linked to the concept of the greater good for society and linked to the concept of virtue ethics;

Some of the particular ethical challenges confronted by the use of AI are:

- Lack of transparency of AI tools: AI decisions are not always intelligible to humans;
- AI is not neutral: AI-based decisions are susceptible to inaccuracies, discriminatory outcomes, embedded or inserted bias;
- Surveillance practices for data gathering and privacy of court users;
- New concerns for fairness and risk for Human Rights and other fundamental values;
- AI will be complementing but not replacing doctors and other healthcare professionals.

With all these topics in mind, there is what is considered a new branch in the field of ethics, known as Data Ethics. It represents the application of moral and ethical principles to solve problems arisen to data (generation, recording, curation, processing, dissemination, sharing and use), algorithms (AI, machine learning and robots) and corresponding practices in order to support and back up morally good solutions, right behaviours and right values^9^. The introduction of AI in many aspects of surgery may be assimilated to the introduction of a new technology, and thus the same recommendations for its use applies.

The four ethical principles guide the ethical behavior in the surgical care setting and provide a framework for the application of surgical ethics. Patients may be at risk when AI tools or capabilities are used. Thus the ethical principles may be applied to the world of AI and its byproducts as follows and providing a framework for solutions when confronted to conflicts in the real world of surgical healthcare and research (Table 1). The principle of beneficence surrounds the issues of: protection and promotion of human health, transparency, quality of data, use and safety of data, adequate management of data to avoid bias or inaccuracies, human rights and values, and trustworthiness. The principle of nonmaleficence plays a role in: reliability, transparency, AI-aided surgical decision-making, algorithmic flaws and black-box systems opacities, cybersecurity, errors in the processes and in final decision-making and/or therapy indication, vulnerability regarding biases, safety, security and opacity and protection of privacy, security, values of patients. The principle of respect for autonomy supports the surgical informed consent process, transparency and truth telling, confidentiality, communication, human autonomy and privacy (in particular to sensitive information), corporate involvement and financial gain, the role of IRBs in research using big data, data breaches. The principle of justice provides support in: transparency, accountability, human rights, threat of medical liability, fairness in the use of data, transparency, prevention of unequities, biases. The concept of transparency is overruled by the four ethical principles, meaning that the actions, processes and data should be open to inspection and assessment in a mandatory fashion added to the explainability and accountability at all stages of AI processes and ML algorithms. There is an inherent complexity of the ethical challenges posed by AI and data science; because of this complexity an ethical concern and thoughtfulness should be developed from the start^10^. The goals of a stringent ethical approach to the development and application of AI in the surgical field are mainly two: the development of a deeply inherent culture of accountability and the adoption of a governance system promoting the adoption of good and ethically sound practices at every point in the innovation and implementation process by all stakeholders.

**Table 1:** Ethical principles and their taxonomy within the realm of AI.

Beneficence	Non-maleficence	Respect for autonomy	Justice
• Protection and promotion of human health	• Reliability	• The surgical informed consent process	• Transparency
• Transparency	• Transparency	• Transparency	• Accountability
• Quality of data	• AI-aided surgical decision-making	• Truth telling	• Human rights
• Use and safety of data	• Algorithmic flaws and black-box systems opacities	• Confidentiality	• Threat of medical liability
• Adequate management of data to avoid bias or inaccuracies	• Cybersecurity	• Communication	• Prevention of inequities
• Human rights and values	• Errors	• Human autonomy and privacy (in particular to sensitive information)	• Biases
• Trustworthiness	• Vulnerability regarding biases, safety, security and opacity	• Corporate involvement and financial gain	
	• Protection of privacy, security, values of patients	• Role of IRBs in research using big data	
		• Data breaches	

These specific steps will help users and researchers to successfully navigate the different stages of the processes involved:

- Define and understand the benefit for society and its greater good;
- Compliance with the ethical principles and their taxonomy as well as with the law regulations;
- Audit of the quality and limitations of the data;
- Permanent assessment and evaluation as well as policy implications.

In summary, it should be recognized that the goal of health applied AI-systems is to contribute to the wellbeing of patients and their safety and quality of care. AI needs to be ethically accountable, recommending frequent audits of algorithms and overall processes. AI will unavoidably be part of our surgical practice in the future, just in a similar fashion to any other field or industry or activity. The gordian knot is not whether AI will influence and change surgical practice, but rather how we can adopt it with stringent ethical principles and normative framework putting the patient at the center of our care and concerns.
