# Erratum

**DOI:** 10.1590/0100-6991e-20243762errata-en

**Published:** 2024-07-05

The article “Ethical aspects of artificial intelligence in general surgical practice”, DOI number: 10.1590/0100-6991e-20243762EDIT01-en, published in the Journal of the Brazilian College of Surgeons 51:e20243762EDIT01.

Where it is:

Mailing address: 

Ramiro Colleoni 

E-mail: rcolleoni@uol.com.br

It should be: 

Mailing address: 

Alberto R Ferreres

E-mail: albertoferreres@gmail.com

